# Dual-time-point myocardial ^18^F-FDG imaging in the detection of coronary artery disease

**DOI:** 10.1186/s12872-017-0554-x

**Published:** 2017-05-10

**Authors:** Ke-Fei Dou, Xiao-Jin Gao, Bo-Qia Xie, Yan Li, Zuo-Xiang He, Min-Fu Yang

**Affiliations:** 1 State Key Laboratory of Cardiovascular Disease, Department of Cardiology, Cardiovascular Institute, Fuwai Hospital and National Center for Cardiovascular Diseases, Chinese Academy of Medical Sciences and Peking Union Medical College, Beijing, China; 2 National Center for Cardiovascular Diseases, Chinese Academy of Medical Sciences and Peking Union Medical College, A 167, Beilishi Road, Xicheng District, Beijing, 100037 China; 3 Department of Cardiology, Beijing Chaoyang Hospital, Capital Medical University, Beijing, China; 4 Department of Nuclear Medicine, Cardiovascular Institute, Fuwai Hospital and National Center for Cardiovascular Diseases, Chinese Academy of Medical Sciences and Peking Union Medical College, Beijing, China; 5 Department of Nuclear Medicine, Beijing Chaoyang Hospital, Capital Medical University, 8th Gongtinanlu Rd, Chaoyang District, Beijing, 100020 China

**Keywords:** Dual-time-point imaging, ^18^F-FDG, Coronary artery disease, Myocardial ischemia

## Abstract

**Background:**

Myocardial ^18^F-deoxyglucose (^18^F-FDG) uptake has been observed to be enhanced in patients with coronary artery disease (CAD) under fasting conditions. However, whether the increased ^18^F-FDG is induced by myocardial ischemia and how to discriminate ischemic from physiological ^18^F-FDG uptake have rarely been investigated.

**Methods:**

Under fasting conditions, ^18^F-FDG PET imaging was performed in 52 patients with suspected CAD. Two ^18^F-FDG imaging sessions were conducted within two hours after a single administration of ^18^F-FDG (dual-time-point imaging), and with an intervention of an exercise test after the first imaging. Abnormal ^18^F-FDG uptake was determined by the classification of the ^18^F-FDG distribution pattern, and the changes of the ^18^F-FDG distribution between the two PET imaging sessions were analyzed. ^99m^Tc-sestamibi was injected at peak exercise and myocardial perfusion imaging (MPI) was conducted after ^18^F-FDG imaging. Coronary angiography was considered the reference for diagnosing CAD.

**Results:**

Overall, 54.8% (17/31) of CAD patients and 36.2% (21/58) of stenotic coronaries showed exercise-induced abnormal uptake of ^18^F-FDG. Based on the classification of the ^18^F-FDG distribution pattern, the sensitivity and specificity of exercise ^18^F-FDG imaging to diagnose CAD was 80.6% and 95.2% by patient analysis, 56.9% and 98.0% by vascular analysis, respectively. Compared with MPI, ^18^F-FDG imaging had a tendency to have higher sensitivity (80.6% vs 64.5%, *P* = 0.06) on the patient level.

**Conclusion:**

Myocardial ischemia can induce ^18^F-FDG uptake. With the classification of the ^18^F-FDG distribution pattern, dual-time-point ^18^F-FDG imaging under fasting conditions is efficient in diagnosing CAD.

## Background

Glucose uptake in normal myocardium is suppressed under fasting conditions, nevertheless myocardial ischemia results in a dramatic switch of metabolic substrate to glucose uptake. Hence, the ischemic and normal myocardium have significant difference in glucose uptake [1, 2]. Therefore, myocardial ^18^F-deoxyglucose (^18^F-FDG) imaging under fasting conditions is in theory a potential method to detect myocardial ischemia. Previous studies have observed enhanced ^18^F-FDG uptake in myocardium supplied by stenotic coronaries or in myocardium with perfusion abnormalities [3–11]. However, whether the enhanced ^18^F-FDG uptake is induced by myocardial ischemia is controversial [12], since non-ischemic myocardium can also exhibit varying extents of ^18^F-FDG uptake even under strict dietary control [13–15]. In the present study, we performed dual-time-point imaging within two hours after a single administration of ^18^F-FDG, and myocardial ischemia was induced using an exercise test after the first imaging. We speculated that most factors that presumably affect myocardial ^18^F-FDG uptake would not significantly change in such a short time, and thus we could evaluate whether exercise-induced myocardial ischemia would induce myocardial ^18^F-FDG uptake.

Moreover, even if myocardial ischemia can induce ^18^F-FDG uptake, another issue that comes along is how to differentiate the ischemic uptake from the uptake in non-ischemic myocardium, which was considered physiological. Several studies have investigated the distribution pattern of ^18^F-FDG in patients without heart disease [11, 16, 17]. These studies found that most individuals had no obvious ^18^F-FDG uptake, or had diffuse but homogenous uptake. Contrarily, ‘focal’ or ‘focal on diffuse’ uptake was rarely observed. Even if some patients showed focal uptake, the uptake was usually located on the basal segments, including the papillary muscle. We had adopted these classification methods to differentiate ischemic from physiological uptake in patients with suspected unstable angina who underwent resting ^18^F-FDG imaging, and obtained favorable diagnostic results [11]. In this study, we evaluated the diagnostic performance of ^18^F-FDG imaging in patients with suspected CAD using the classification of ^18^F-FDG distribution pattern.

## Methods

### Study population

This prospective study was approved by the Institutional Ethics Committee of Fuwai Hospital, and written consent was obtained from all patients before study entry. Fifty-five patients with suspected stable angina (37 patients) or unstable angina (18 patients) were recruited. Patients with suspected unstable angina were simultaneously included in the other study aiming at investigating the value of ^18^F-FDG imaging in the diagnosis of unstable angina [11]. Angina was well controlled in all patients and no new onset of angina occurred for > 40 h before the entry to the present study. Patients with the following conditions were not recruited: prior myocardial infarction (history of previously documented myocardial infarction or Q waves on electrocardiogram), acute myocardial infarction, unable to exercise, prior coronary revascularization of less than 3 months, left bundle branch block, diagnosed valvular heart disease, or idiopathic cardiomyopathy.

### Study protocol

**Fig. 1:**
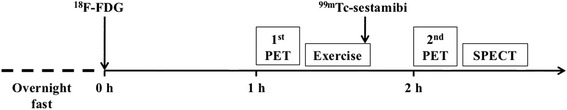
Schematic of imaging protocols

Figure 1 shows the flow diagram of the study protocol. Enrolled subjects were asked to have an overnight fast (>12 h). The imaging studies were arranged in the morning, and anti-angina medications were withheld for > 12 h before exercise testing. Patients first had their blood glucose levels measured using finger blood samples, and then were intravenously injected with ^18^F-FDG (5–8 mCi) at rest. Thereafter, patients underwent two separate PET imaging sessions with a single dose of ^18^F-FDG. This imaging protocol was named as dual-time-point imaging. Briefly, patients underwent the first ^18^F-FDG imaging (rest) using PET/CT one hour after ^18^F-FDG injection. After the first imaging session, they immediately underwent a symptom-limited exercise test using a bicycle ergometer with continuous electrocardiogram (ECG) and blood pressure monitoring. ^99m^Tc-sestamibi (20–25 mCi) was intravenously injected at peak exercise. Approximately two hours after ^18^F-FDG injection, the second ^18^F-FDG imaging (exercise) was performed.

Exercise ^99m^Tc-sestamibi myocardial perfusion imaging (MPI) was done after the second ^18^F-FDG imaging using SPECT. Resting MPI was completed within 3 days of exercise imaging.

### Image acquisition and reconstruction

^18^F-FDG images were acquired, using a Biograph 64 PET/CT scanner (Siemens Medical Solutions, Knoxville, TN, USA) equipped with high-performance LSO PET crystals and a 64-slice CT. After a scout CT acquisition (120 kV, 10 mA), used for appropriate patient positioning, a CT transmission scan (140 kV, 35 mA) was performed for attenuation correction and anatomical localization. PET images were then acquired in list mode with a static 10-min frame. Raw images of ^18^F-FDG were reconstructed by iterative ordered subset expectation maximization (OSEM) reconstructions (8 subsets, 4 iterations) and automatically corrected for photon attenuation using the CT scans.

Exercise ^99m^Tc-sestamibi images were obtained as previously described using a dual-head, large-field-of-view SPECT (Infinia; GE Healthcare, Milwaukee, Wisconsin, USA), equipped with ultra-high-energy parallel-hole collimators [9, 11]. Resting ^99m^Tc-sestamibi images were obtained using the same SPECT with ultra-high-energy parallel-hole collimators or low-energy high-resolution parallel-hole collimators. Thirty projection images were acquired over a 180° arc at 6° intervals. Perfusion images were reconstructed using standard filtered back projection.

### Image analysis

^18^F-FDG and ^99m^Tc-sestamibi images were analyzed separately. Two experienced nuclear physicians interpreted the images independently, and disagreements were resolved by consensus.

#### ^18^F-FDG

^18^F-FDG images were first semi-quantitatively analyzed using standard uptake value (SUV). The left ventricle was divided into 17 segments using the standard American Heart Association model, and each segment was further assigned to three major coronary territories. The maximal SUV of each segment (SUV_myo_) was manually measured guided by CT and fusion images. The SUV of each coronary was represented by the highest segmental SUV_myo_ within its territory. Furthermore, a ROI of 3.0 cm^3^ was placed over the LV chamber near the mitral valve plane to measure the SUV_blood_. Each pair of ROIs on the rest and exercise images was carefully placed on the same region.

Thereafter, the myocardial^18^F-FDG uptake of left ventricle as a whole was classified into four patterns, based on the results of semi-quantitative analysis as well as visual observation [11, 16, 17]: the ‘none’ pattern, indicated the SUV_myo_ in all segments was below or equal to the SUV_blood_; the ‘diffuse’ pattern, indicated the SUV_myo_ in all segments was higher than the SUV_blood_, nonetheless no significant difference existed among them (i.e., homogenous uptake); the ‘focal’ pattern, indicated that at least one segment had a SUV_myo_ higher than the SUV_blood_ and the SUV_myo_ in other segments was below or equal to the SUV_blood_; and the ‘focal on diffuse’ pattern, indicated the SUV_myo_ in all segments was higher than the SUV_blood_, and even higher uptake was identified in one or more segments. Finally, focal uptake in the ‘focal’ or ‘focal on diffuse’ pattern was defined as abnormal, except those on the basal segments and papillary muscle, which were considered normal [18]. Moreover, the changes of ^18^F-FDG distribution pattern between exercise and resting images were analyzed.

#### ^99m^Tc-sestamibi

^99m^Tc-sestamibi images were scored using the same 17-segment model, but with a 5-score scale (0 = normal uptake, 1 = mild, 2 = moderate and 3 = severe reduction in uptake, and 4 = no uptake). Exercise–rest perfusion defects were classified as fixed, reversible, or partially reversible. Abnormal ^99m^Tc-sestamibi uptake was further assigned to three major coronary territories.

### Coronary angiography

Coronary angiography within 3 months of scintigraphic imaging was analyzed. Angiographic results were reviewed for the presence, localization, and severity of coronary artery lesions. Luminal diameter narrowing of ≥ 50% in any of the major epicardial coronary arteries was considered significant and was defined as CAD.

### Statistical analysis

Statistical analyses were performed using SPSS software (version 17.0, SPSS, Inc, Chicago, IL, USA). Continuous variables were described as means and standard deviations (SD) or medians and interquartile ranges, depending on the normality of distribution assessed using the Kolmogorov-Smirnoff test. Categorical variables were described as numbers and percentage. Paired *t* test or Wilcoxon rank test was used to compared the differences of SUVs between resting and exercise ^18^F^−^FDG imaging, depending on the normality of the distribution. McNemar’s test was used to compare the differences of the diagnostic performance between perfusion and ^18^F^−^FDG imaging, and to compare the differences of the prevalence rate of ^18^F-FDG abnormalities between resting and exercise imaging. The variables that potentially influenced the diagnostic sensitivity of ^18^F-FDG imaging, were analyzed using χ^2^ test. A *P* value of < 0.05 was considered statistically significant.

## Results

### Patient characteristics

Of 55 patients initially recruited, 3 cases without coronary angiography were excluded from the final analysis. The demographic, angiographic, and exercise testing data of the remaining 52 patients are listed in Table 1. Of them, 54% were men and the average age was 58 ± 8 years. Ten subjects (19%) had a history of percutaneous coronary intervention (>3 months). Coronary angiography showed significant coronary stenosis in 58 coronaries of 31 patients. Of them, 1-, 2- and 3-vessel disease was 42%, 29%, and 29%, respectively. The mean exercise time was 461 ± 169 s, and the rate-pressure product increased from (7.9 ± 1.9) x 10^3^ at baseline to (22.0 ± 6.5) x 10^3^ at peak exercise. During exercise, 32% of the patients had chest pain and 48% of the patients had ischemic ECG changes.

**Table 1:** Patient characteristics

Characteristics	Results
Age (years)	58 ± 8
Male (%)	28 (54)
Diabetes mellitus (%)	22 (42)
Hypertension (%)	38 (73)
Hypercholesterolemia (%)	32 (62)
Smoker (%)	21 (40)
Family history of CAD	7 (14)
Previous PCI (%)	10 (19)
Blood glucose (mmol/L)	6.7 ± 1.5
Coronary angiography (%)	
One-vessel disease	13 (42)
Two-vessel disease	9 (29)
Three-vessel disease	9 (29)
Exercise test	
Exercise time (s)	461 ± 169
Baseline RPP	7900 ± 1900
Peak RPP	22000 ± 6500
Chest pain (%)	17 (32)
Positive ECG results (%)	25 (48)

*CAD* coronary artery disease, *PCI* percutaneous coronary intervention; and RPP, rate pressure product

### Myocardial perfusion imaging

In total, 20 CAD patients had perfusion abnormalities. Of them, 6 patients had partially reversible perfusion defects, and 14 had reversible perfusion defects. No patients without CAD showed perfusion abnormality. Therefore, the sensitivity and specificity of exercise MPI in the diagnosis of CAD was 64.5% and 100%, respectively. By vascular analysis, the sensitivity and specificity was 53.4% and 99.0%, respectively (Table 2).

**Table 2:** Diagnostic performance of exercise perfusion and ^18^F-FDG imaging

	Patient analysis		Vascular analysis	
	Sensitivity	Specificity	Sensitivity	Specificity
^99m^Tc-sestamibi	64.5% (20/31)	100% (21/21)	53.4% (31/58)	99.0% (97/98)
^18^F-FDG	80.6% (25/31)	95.2% (20/21)	56.9% (33/58)	98.0% (96/98)
*P* value	0.06	1.00	0.79	1.00

### ^18^F-FDG imaging

#### Changes of ^18^F-FDG uptake on dual-time-point imaging

**Fig. 2:**
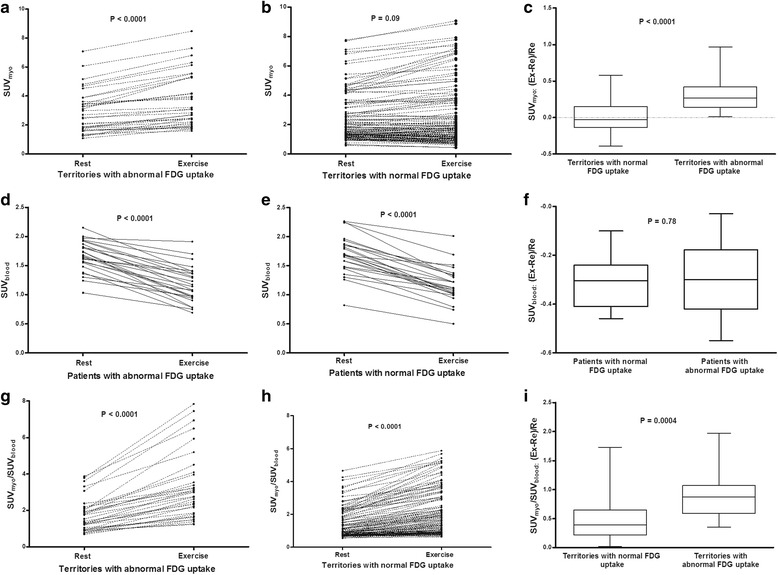
SUV changes of ^18^F-FDG uptake on dual-time-point imaging. All coronary territories with abnormal ^18^F-FDG uptake show an increase in SUV_myo_ on exercise imaging (**a**). Contrarily, only 44.6% of coronaries with normal ^18^F-FDG uptake show increased uptake (**b**). Therefore, the increment in territories with abnormal ^18^F-FDG uptake is significant higher than that in normal territories (**c**). The SUV_blood_ consistently decreases from rest to exercise imaging in patients with abnormal (**d**) and normal ^18^F-FDG uptake (**e**), and the reduction is similar between the two groups (**f**). As a result, although both abnormal and normal territories show an increase in SUV_myo_/SUV_blood_ (**g, h**), the increment is higher in territories with abnormal ^18^F-FDG uptake (**i**)

By semi-quantitative analysis, all coronary territories with abnormal ^18^F-FDG uptake showed an increase in SUV_myo_ on exercise imaging, and that was only 44.6% in coronaries with normal ^18^F-FDG uptake (Fig. 2a-c). Meanwhile, the SUV_blood_ was decreased on exercise imaging in all patients (Fig. 2d-f). Consequently, the SUV_myo_/SUV_blood_ consistently increased from rest to exercise ^18^F-FDG imaging in all coronaries, nevertheless, coronaries with abnormal ^18^F-FDG uptake had a higher increment (Fig. 2g-i).

**Fig. 3:**
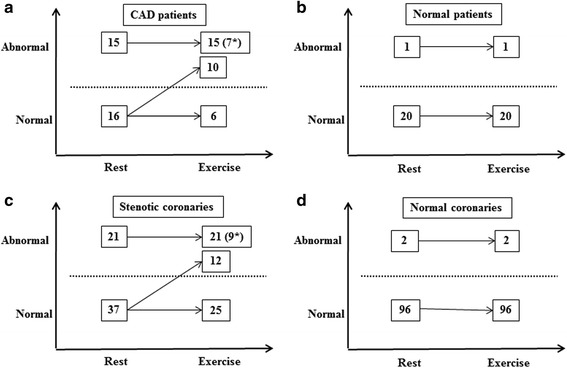
Changes of ^18^F-FDG distribution characteristics between resting and exercise imaging. By patientanalysis, the prevalence rate of ^18^F-FDGabnormalities increases from 48.4% (15/31) on resting imaging to80.6% (25/31) (*P* = 0.004) on exercise imaging in CAD patient (**a**) nevertheless it does not significantly change in patients without CAD [4.8% (1/21) vs 4.8% (1/21) P = NS] (**b**) By vascular analysis, the prevalence rate of ^18^F-FDG abnormalities on exercise imaging is significantly higher than that on resting imaging in stenotic coronaries [56.9% (33/58) vs 34.5% (21/58), *P* < 0.0001] (**c**) but not in normal coronaries [2.0% (2/98) vs 2.0% (2/98), *P* = NS] (**d**). (*)*Indicates the increased* ^*18*^ *F-FDG uptake is exclusively located on the basal segments or papillary muscle*

**Fig. 4:**
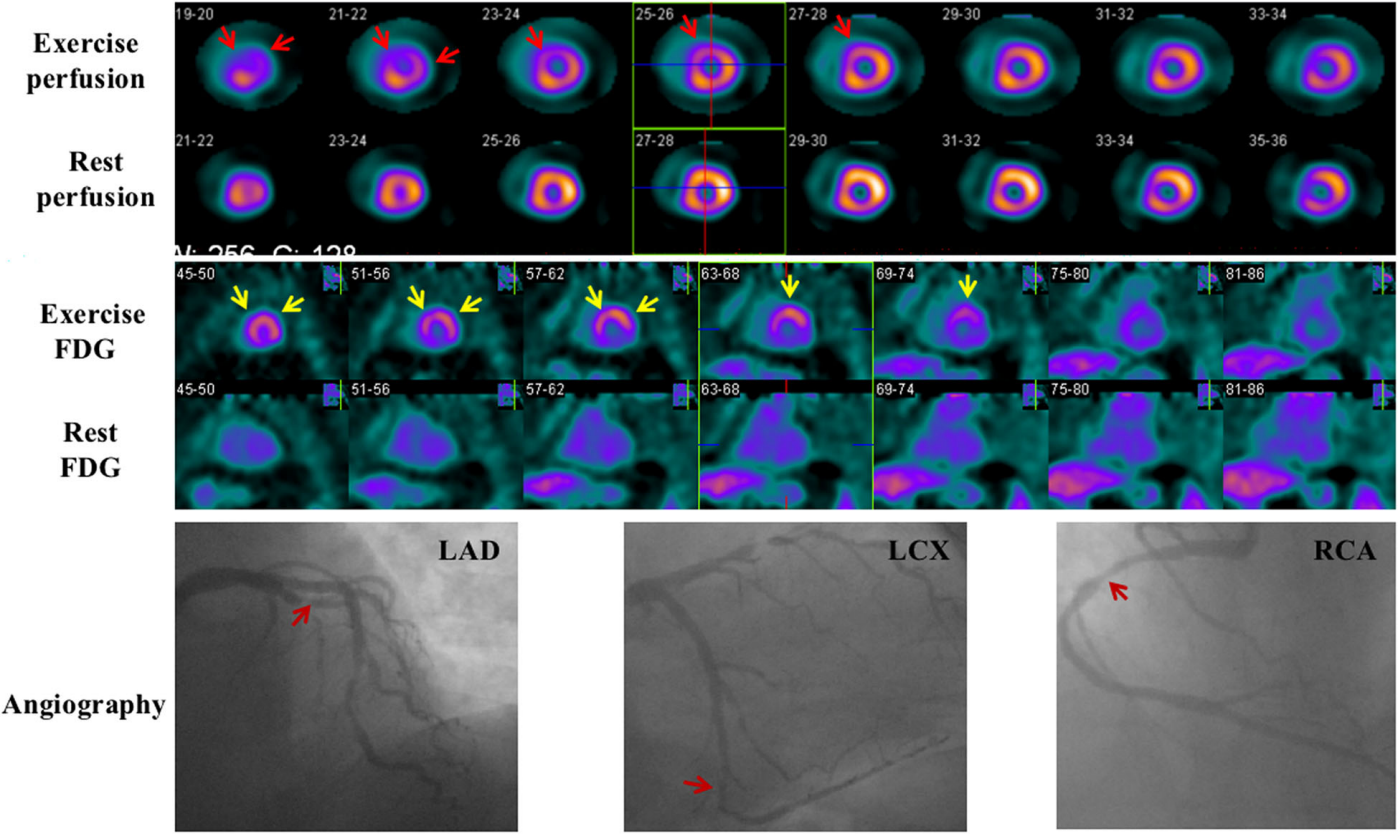
Images of a 61-year-old woman. She had a 99% stenosis in the left anterior descending coronary (LAD), a 90% stenosis in the left circumflex coronary (LCX), and a 70% stenosis in the right coronary artery (RCA). Perfusion images show reversible defects in the anterior and lateral wall (*red arrows*). Resting ^18^F-FDG images indicate only background uptake in the cardiac cavities and no visible uptake in the left ventricular wall (‘none’ pattern). Nevertheless, exercise ^18^F-FDG images exhibit intense uptake in the anterior and lateral wall (‘focal’ pattern, *yellow arrows*) which is in accordance with exercise perfusion images

**Fig. 5:**
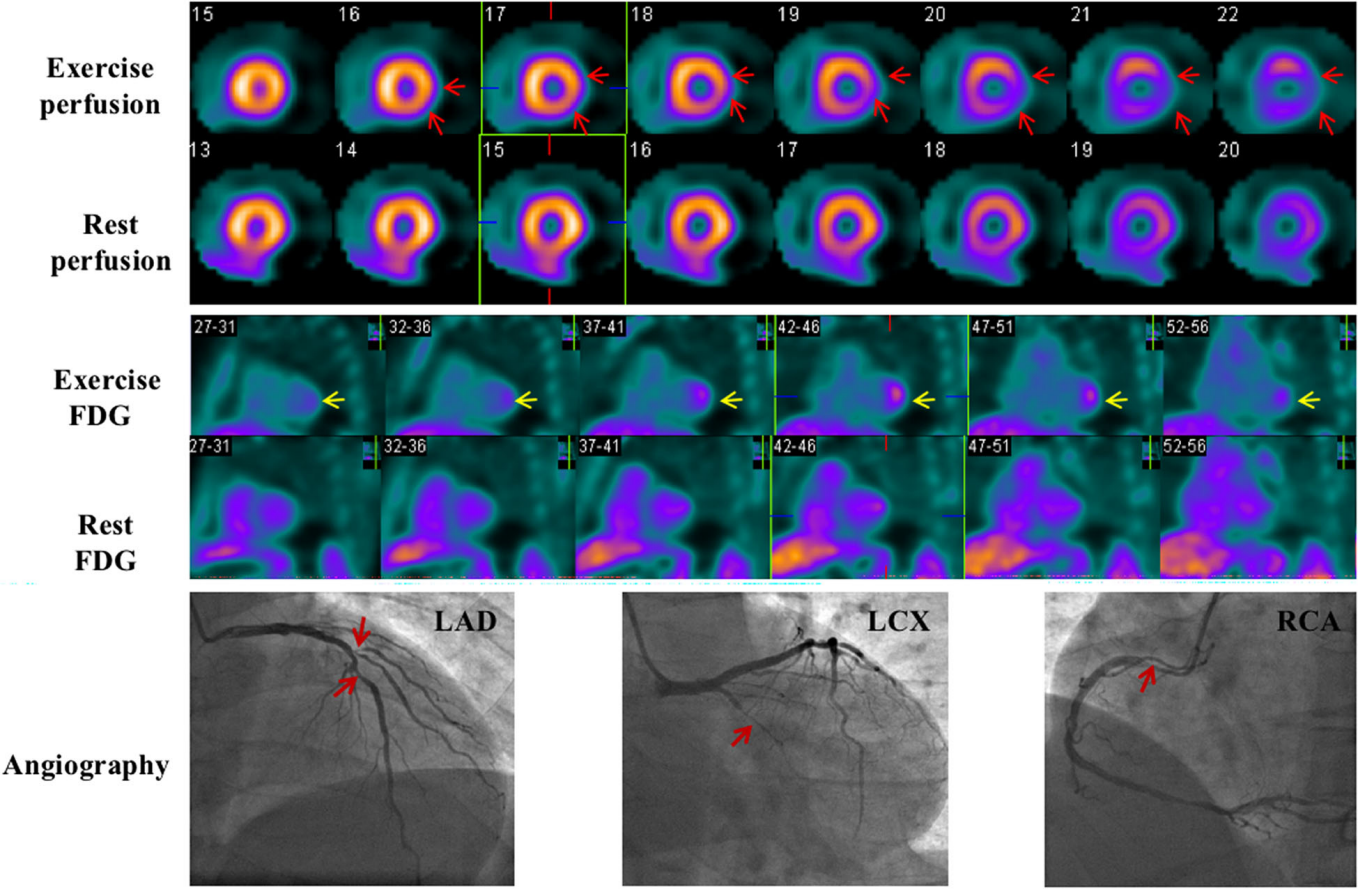
Images of a 39-year-old man. He had a 70% stenosis in the LAD and a 90% stenosis in the diagonal branch, a 100% stenosis in the LCX, and a 90% stenosis in the proximal RCA. Perfusion images show reversible defects in the lateral wall (*red arrows*). Resting ^18^F-FDG images indicate background uptake in the cardiac cavities and no visible uptake in the left ventricular wall (‘none’ pattern), exercise ^18^F-FDG images exhibit intense uptake in the lateral wall (‘focal’ pattern, *yellow arrows*) which is in accordance with exercise perfusion images

**Fig. 6:**
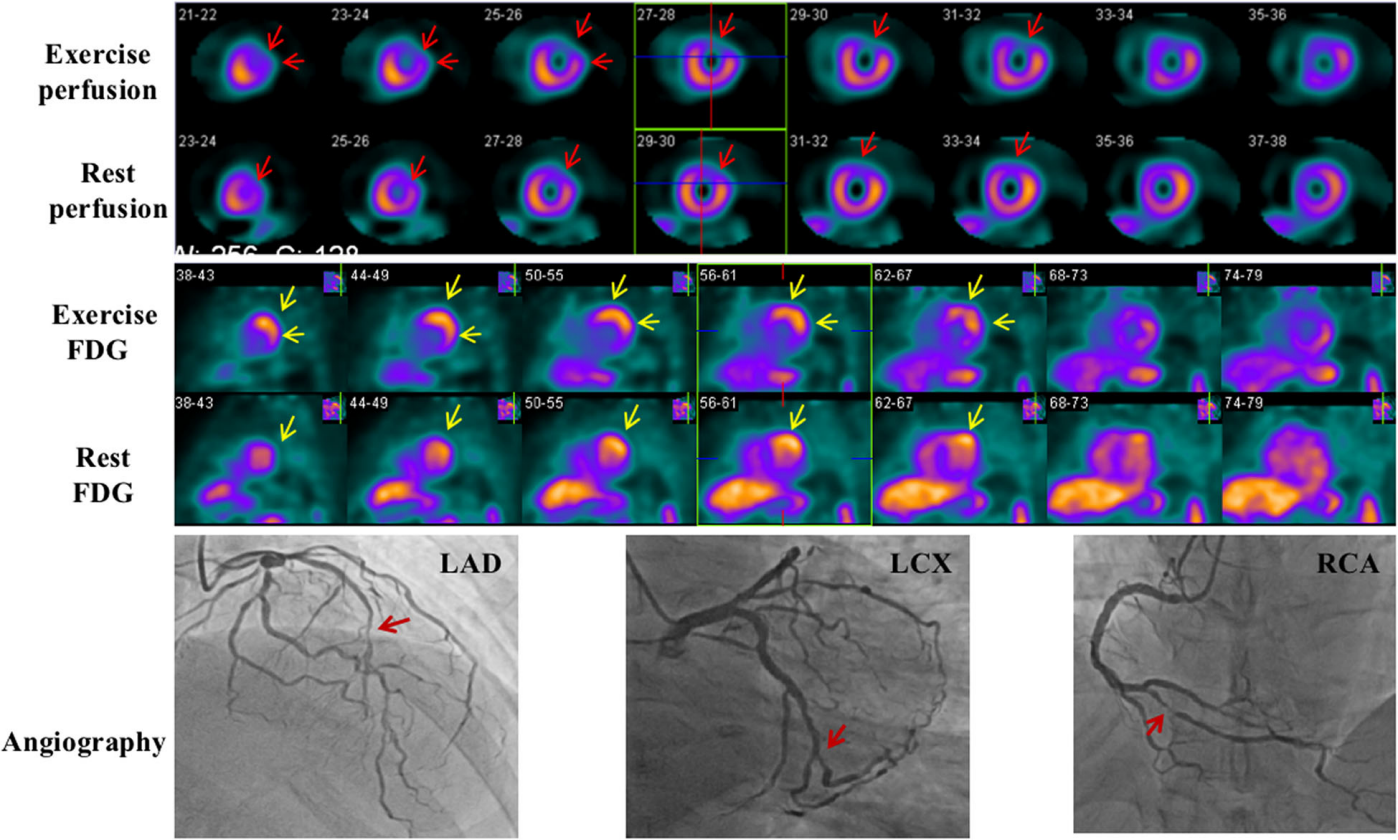
Images of a 55-year-old woman. She had multiple lesions of > 90% stenosis in the LAD, multiple lesions of 60-80% stenosis in the LCX, and a distal lesion of 90% stenosis in the RCA. Perfusion images show partially reversible defects in the anterior and anterolateral wall (‘focal’ pattern, *red arrows*). Resting ^18^F-FDG images show mild uptake in the anterior and anterolateral wall. On exercise images, ^18^F-FDG uptake is further increased compared with resting images, and the involved area is enlarged as well (‘focal’ pattern, *yellow arrows*)

By qualitative analysis, 10 CAD patients had abnormal ^18^F-FDG uptake on exercise imaging but normal uptake on resting imaging (Fig. 3a-b, Figs. 4 and 5). Fifteen patients had abnormal uptake on both imaging, but 7 of them had more myocardial segments involved on the exercise imaging (Fig. 6). Of the stenotic coronaries, 12 showed abnormal ^18^F-FDG uptake exclusively on exercise imaging. Twenty-one stenotic coronaries showed abnormal uptake on both imaging, but 9 of them had more myocardial segments involved on the exercise imaging (Fig. 3c-d). In sum, 54.8% (17/31) of CAD patients and 36.2% (21/58) of stenotic coronaries exhibited exercise-induced ^18^F-FDG changes.

#### Diagnostic performance of ^18^F-FDG imaging

By patient analysis, abnormal ^18^F-FDG uptake was present on exercise imaging in 26 patients (25 with CAD and 1 without CAD). The sensitivity and specificity of exercise ^18^F-FDG imaging in the diagnosis of CAD was 80.6% (25/31) and 95.2% (20/21) (Table 2), respectively. Compared with MPI, ^18^F-FDG imaging had a tendency to have higher sensitivity (80.6% vs 64.5%, *P* = 0.06), but similar specificity (95.2% vs 100%, *P* = 1.00).

By vascular analysis, abnormal ^18^F-FDG uptake was present on exercise imaging in 35 coronary territories (33 with and 2 without significant stenosis). The sensitivity and specificity of exercise ^18^F-FDG imaging in the detection of stenotic coronaries were 56.9% (33/58) and 98.0% (96/98) (Table 2), respectively. Both the sensitivity and specificity of exercise ^18^F-FDG imaging were not significantly different from that of MPI (sensitivity: 56.9% vs 53.4%, *P* = 0.79; specificity: 98.0% vs 99.0%, *P* = 1.00).

#### Variables related to the diagnostic performance of ^18^F-FDG imaging

**Fig. 7:**
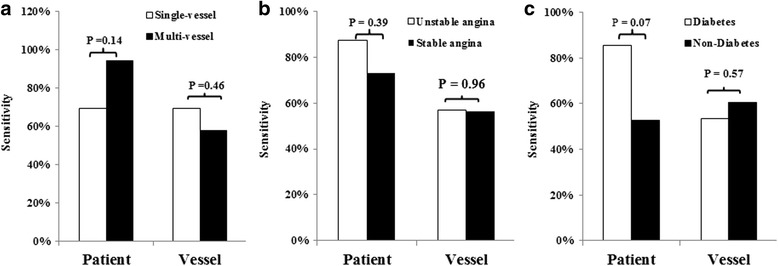
Variables influence the diagnostic sensitivity of ^18^F-FDG imaging. The sensitivity of single-vessel and multivessel disease was comparable at both patient level and individual vessel level (**a**) The difference of sensitivity is not significant between stable and unstable angina (**b**) and is borderline on the patient level but not on the vascular level between diabetic and non-diabetic patients (**c**)

The sensitivity of single-vessel and multi-vessel disease was comparable at both patient level (69.2% vs 94.1%, *P* = 0.14) and individual vessel level (69.2% vs 57.8%, *P* = 0.46) (Fig. 7a).

The sensitivity was not significantly different between patients with suspected stable angina and unstable angina both on patient and vascular level (Fig. 7b).

Diabetic patients had higher blood glucose than non-diabetes (7.6 ± 0.3 mmol/l vs 5.9 ± 0.1 mmol/l, *P* < 0.0001). Compared with normal subjects, diabetic patients had similar SUV_blood_ (1.66 ± 0.20 vs 1.70 ± 0.35, *P* = 0.59), but lower SUV_myo_ (1.62 ± 0.97 vs 2.78 ± 1.78, *P* = 0.0027) and SUV_myo_/SUV_blood_ (1.19 ± 0.72 vs 1.99 ± 1.28, P = 0.0032) on resting imaging. ^18^F-FDG imaging had a trend to have higher sensitivity for diabetic patients on the patient level (*P* = 0.07, Fig. 7c) but not on the vascular level (*P* = 0.57) (Fig. 7c).

## Discussion

By the direct comparison of the images obtained before and after an exercise test within two hours, the present study demonstrated a significant change of ^18^F-FDG uptake in CAD patients and stenotic coronaries. Previous studies have reported enhanced ^18^F-FDG uptake in patients with CAD under fasting conditions [3–11]. However, myocardial ^18^F-FDG uptake is spatially heterogeneous even under strict dietary control [13–15]. Patients without documented CAD can also have regional or global myocardial uptake, which is known as ‘non-specific’ or ‘physiological’. Physiological uptake is mostly localized on, but not confined to, the basal segments. Therefore, regional ^18^F-FDG uptake, even in the territory supplied by the stenotic coronary, may only be physiological. As a result, although ^18^F-FDG imaging had a relatively high sensitivity in the diagnosis of CAD, its specificity was rarely investigated and presumably poor [10]. To clarify the specificity of ^18^F-FDG imaging, we had conducted exercise and resting ^18^F-FDG imaging in CAD patients during two sequential days [9]. Resting imaging was 24 h after exercise imaging. We speculated that the ischemic uptake of ^18^F-FDG on exercise imaging should decrease or disappear on resting imaging. In that study, 87% of the patients with increased ^18^F-FDG uptake on exercise imaging showed decreased or no discernible uptake on resting imaging, which in part supported that ^18^F-FDG uptake was a specific marker for myocardial ischemia. The other 13% patients had persistent uptake which was interpreted as ischemic memory. However, several studies have found that normal myocardium could also show a significant change in ^18^F-FDG uptake among serial examinations [14, 15], which was considered as temporal heterogeneity. A number of known factors (hormones, dietary preparation, exercise, etc.) and unknown factors might influence myocardial ^18^F-FDG uptake, and it was difficult to modulate these conditions to the same level on different days. Contrary to the prior studies [14, 15], the two ^18^F-FDG imaging sessions in this study were performed within a very short interval of two hours, and only with a single administration of ^18^F-FDG. This could, to the greatest extent, alleviate the differences of the aforementioned factors between exercise and resting imaging. Catecholamines released during exercise stress suppress exogenous glucose metabolism in normal myocardium but do not alter anaerobic glucose metabolism in ischemic myocardium [12, 19], but no study has reported that that effect was spatially heterogeneous. Hence, the regionally increased uptake on exercise imaging in CAD patients was most likely induced by myocardial ischemia. The significance of the present study was, for the first time, providing unequivocal evidence in support of that myocardial ischemia can induce ^18^F-FDG uptake.

On the exercise imaging, the ^18^F-FDG uptake in ischemic myocardium was consistently increased. Contrarily, non-ischemic myocardium showed variable changes of ^18^F-FDG uptake and nearly half of them had decreased uptake. Moreover, the SUV_blood_ consistently decreased on the exercise imaging both in CAD and normal patients. Therefore, the ^18^F-FDG distribution changes between resting and exercise imaging was due to the increased uptake ratio of ischemic to non-ischemic myocardium as well as the myocardium to background radioactivity.

Of note, some patients showed ischemic ^18^F-FDG uptake on resting and nearly half (7/15) of them had more segments involved on exercise imaging. Abnormal ^18^F-FDG uptake at rest may be induced by resting ischemia since 6 patients had perfusion abnormalities at rest in this study. Moreover, patients with unstable angina may have abnormal ^18^F-FDG uptake at rest due to ischemic memory, even without perfusion abnormalities, which had been validated in prior studies [11, 20].

To determine the abnormality of ^18^F-FDG uptake, we adopted the classification of ^18^F-FDG distribution patterns and defined the uptake of basal segments and papillary muscle as normal, as recommended recently for the ‘hot’ spot imaging for the heart [18]. This study demonstrated this strategy was efficient for the detection of CAD. Overall, the diagnostic performance was comparable between ^18^F-FDG imaging and MPI. Especially, a favorable specificity was obtained on both patient and vascular levels. In this study, all patients with perfusion abnormalities showed abnormal ^18^F-FDG uptake on exercise imaging. Furthermore, 45.5% (5/11) of CAD patients with normal perfusion were detected by ^18^F-FDG imaging as well. This implied that ^18^F-FDG imaging may be more sensitive than MPI. It is important to note, the difference of the sensitivity between ^18^F-FDG imaging and MPI was merely borderline (*P* = 0.06) on the patient level. In contrast, previous studies have reported higher sensitivity of ^18^F-FDG imaging over MPI [7, 10]. There were two major differences between the present and prior studies. First, ^18^F-FDG was administered at peak exercise in previous studies. Contrarily, ^18^F-FDG was injected one hour prior to exercise in the present study. Due to the extraction by myocardium and other tissues, and clearance from urinary and digestive system, ^18^F-FDG in blood at the onset of exercise-induced ischemia was decreased. In addition, if physiological uptake of myocardium prior to exercise was too intense, new ischemic uptake might be masked. Second, only focally increased ^18^F-FDG uptake was defined as abnormal in this study, whereas diffuse but homogeneous uptake and basal (including papillary muscle) uptake were also considered abnormal in prior studies [3–11]. The current definitions inevitably decreased the sensitivity, but rendered an improved specificity. Recent studies have demonstrated that most individuals without heart disease showed a ‘none’ or ‘diffuse’ pattern, ‘focal’ or ‘focal on diffuse’ uptake was mostly observed in unstable angina [11, 16]. These findings suggested that the pattern, rather than the extent, of ^18^F-FDG uptake, is an indicator of myocardial involvement under fasting conditions. Therefore, we adopted these classifications in the present study. For the same reason, we did not consider quantitative evaluation of ^18^F-FDG uptake as the diagnostic criteria.

The secretion of insulin is impaired and/or there is insulin resistance for diabetic patients. Therefore, the utilization of glucose is decreased in these subjects. However, recent studies have proposed that ischemia and insulin would trigger independent pathways of the translocation of glucose transporters (GLUTs) in the myocardium, to increase glucose transport to the myocyte: ischemia leads to GLUT-4 translocation via a phosphatidylinositol 3-kinase (PI3-kinase)-independent mechanism, and insulin via a PI3-kinase-mediated pathway [21, 22]. These differential regulations of GLUT-4 translocation suggest that even in diabetic patients who have myocardial insulin resistance, would have increased glucose uptake when triggered by ischemic events. Moreover, due to the lower ^18^FDG uptake in non-ischemic myocardium demonstrated in this study, ^18^FDG myocardial ischemic imaging may have a higher specificity in diabetic patients than that in non-diabetic patients.

There are some limitations in this study. This study included a subgroup of patients with suspected unstable angina, who had a higher incidence and more severity of perfusion abnormalities on both resting and exercise imaging. This may result in the overestimation of the sensitivity of ^18^F-FDG imaging. However, we did not yet find significant differences in the diagnostic performance between stable and unstable angina (Fig. 7b). ^18^F-FDG was only injected once and one hour prior to the exercise test in this study. This may, as discussed above, lower the sensitivity of ^18^F-FDG imaging. Whether split dose (i.e., the ^18^F-FDG was divided into 2 parts, and was administered separately at rest and exercise testing) or one injection after exercise followed by two imaging session, can improve the sensitivity needs to be further studied. We only examined the glucose level at the baseline and no other hormones were checked. The difference of these hormones between resting and exercise imaging, and their effects on the myocardial uptake of ^18^F-FDG, could not be evaluated. Exercise perfusion images were acquired using SPECT equipped with ultra-high-energy collimators, which might have underestimated the sensitivity of MPI compared using SPECT equipped with low-energy collimators. Moreover, down-scatter of ^18^F to the ^99m^Tc window might bring quantitation error. The ratio of ^99m^Tc to ^18^F was more than 4–5 : 1 in this study, and the contribution of ^18^F was estimated to be less than 5% of the total counts in the ^99m^Tc window [23]. In addition to fasting, several other strategies have recently been developed to suppress the physiological uptake of ^18^F-FDG in myocardium [24]. However, the application of these interventions in ischemic ^18^F-FDG imaging was scarce and required further investigation [10]. Hence, we did not integrate them into our study protocol. We didn’t integrate functional evaluation of wall motion into the study protocol, hence we couldn’t correlate the metabolic abnormality with myocardial hypokinesis. Therefore, we couldn’t decide in which patient myocardial stunning was the underlying mechanism of metabolic abnormality. Finally, since this was a small and exploratory study, larger studies are necessary.

## Conclusion

The present study demonstrated that myocardial ischemia can induce ^18^F-FDG uptake, and support the potential use of fasting ^18^F-FDG imaging in detecting myocardial ischemia. As no satisfactory methods can completely suppress physiological uptake in non-ischemic myocardium so far, classification of the ^18^F-FDG distribution pattern is an effective alternative in differentiating abnormal ^18^F-FDG uptake.
